# Lessons in Nursing

**Published:** 1889-06

**Authors:** 


PRACTICAL LESSONS IN NURSING.—Diseases and Injuries of the Ear; their Prevention and Cure. By Charles Henry Burnett, M. D., Aural Surgeon to the Presbyterian Hospital; one of the Consulting Aurists to the Pennsylvania Institution for the Deaf and Dumb. Lecturer on Otology, Woman's Medical College of Pennsylvania in Philadelphia; ex-President of the American Otological Society; Fellow of the College of Physicians of Philadelphia. Philadelphia: J. B. Lippincott Company, 1889. Price $1.00.

We have here a well written work of 154 pages, in which the author has succeeded in presenting the Diseases and Injuries of the Ear in a form free from technical terms, and so plain and concise that the ordinary practitioner can understand and avail himself of the many valuable suggestions therein contained.

The author remarks that "The aim has been to show the inexpert what to avoid in the treatment of ear diseases, rather than what they may try to do for their relief."

